# Supplementary material for: Content-rich biological network constructed by mining PubMed abstracts
Source: BMC Bioinformatics. 2004 Oct 8;5:147. doi: 10.1186/1471-2105-5-147 (PMC528731; doi:10.1186/1471-2105-5-147)
Supplement: Additional File 2 — The original results of the above study (non-essential files are deleted to keep the file size under the limit set by BMC bioinformatics). [file 1471-2105-5-147-S2.bz2 › chilibotAdditionalFile2/dip10/333ID8171323E3969/html/TNF_LTBR.html]

 


 **TNF** and **LTBR** 
  
Found 627 abstracts in PubMed, retrieved 10.  
 

 What does Google say? 
 PDF only 
| .edu only 

---

**Interactive relationship** (e.g. stimulation, inhibition, etc)

**Neutral relationship**- LIGHT is a new member of the tumor necrosis factor  [ **TNF** ]  superfamily, which binds to lymphotoxin beta receptor  [ **LTBR** ] , herpes virus entry mediator, or TR6.  Ref: 12767529 Cancer Lett, 2003
- LT consists of soluble LT alpha 3, which binds to  **TNF**  R1 and  **TNF**  R2, and membrane LT alpha 1 beta 2, which binds to LT beta R  [ **LTBR** ] .  Ref: 12882833 Int Immunol, 2003
- LIGHT is a tumor necrosis factor  [ **TNF** ]   **TNF**  superfamily ligand that regulates T cell immune responses by signaling through the herpes virus entry mediator HVEM and the lymphotoxin beta receptor  [ **LTBR** ]  LTbetaR.  Ref: 12787566 Cytokine Growth Factor Rev, 2003

**Non-interactive relationship** (e.g. studied together, co-existance, homology, etc.)

- Isolated Lymphoid Follicle Formation Is Inducible and Dependent Upon Lymphotoxin Sufficient B Lymphocytes, Lymphotoxin beta Receptor  [ **LTBR** ] , and  **TNF**  Receptor I Function.  Ref: 12759424 J Immunol, 2003
- After treatment, the UAER, CD11b CD18  [ **LTBR** ]  expression and  **TNF**  alpha  [ **TNF** ]  concentration lowered significantly in the treated group P <.  Ref: 12872395 Zhongguo Zhong Xi Yi Jie He Za Zhi, 2003
- The changes of urinary albumin excretion rate UAER, CD11b CD18  [ **LTBR** ]  expression and tumor necrosis factor alpha  [ **TNF** ]   **TNF**  alpha  [ **TNF** ]  concentration before and after treatment were observed.  Ref: 12872395 Zhongguo Zhong Xi Yi Jie He Za Zhi, 2003
- Gene knockout study suggests that NIK is dispensable for  **TNF**  R1 but required for lymphotoxin beta receptor  [ **LTBR** ]  induced NF kappaB activation.  Ref: 12853971 Oncogene, 2003
- This activity of LT depends on membrane LT alpha 1 beta 2 and not soluble LT alpha 3, since DA virus persisted and induced inflammatory demyelination in LT beta R  [ **LTBR** ] , but not  **TNF**  R1 or  **TNF**  R2, mice.  Ref: 12882833 Int Immunol, 2003
- The samples were analyzed for red blood cell count, leukocyte count, platelet count, platelet membrane expression of glycoproteins GP Ib and GPIIb IIIa, leukocyte membrane expression of cluster of differentiation CD 11b CD18  [ **LTBR** ] , as well as plasma concentration of tumor necrosis factor  [ **TNF** ]   **TNF**  alpha  [ **TNF** ] , interleukin IL 1beta, and IL 8.  Ref: 12780512 Artif Organs, 2003
- RESULTS The CD11b CD18  [ **LTBR** ]  expression and  **TNF**  alpha  [ **TNF** ]  concentration in DM patients were higher than those of normal range P <.  Ref: 12872395 Zhongguo Zhong Xi Yi Jie He Za Zhi, 2003
- Glands that received AdTNFRp55 Ig concurrently with activated PBLs had decreased numbers of CD4 cells, CD18  [ **LTBR** ]  cells, RTLA, and apoptotic cells.CONCLUSIONSIn vivo transduction of the lacrimal gland with AdTNFRIp55 Ig resulted in transient expression in the gland and the appearance of  **TNF**  inhibitor protein in tears.  Ref: 12792478 Cornea, 2003
